# Combined Stereotactic Body Radiotherapy and Checkpoint Inhibition in Unresectable Hepatocellular Carcinoma: A Potential Synergistic Treatment Strategy

**DOI:** 10.3389/fonc.2019.01157

**Published:** 2019-11-12

**Authors:** Chi-Leung Chiang, Albert C. Y. Chan, Keith W. H. Chiu, Feng-Ming (Spring) Kong

**Affiliations:** ^1^Department of Clinical Oncology, University of Hong Kong, Hong Kong, Hong Kong; ^2^Department of Surgery, University of Hong Kong, Hong Kong, Hong Kong; ^3^Department of Diagnostic Radiology, University of Hong Kong, Hong Kong, Hong Kong; ^4^Department of Radiation Oncology, University Hospitals Cleveland Medical Center, Case Western Reserve University Medical School, Case Comprehensive Cancer Center, Cleveland, OH, United States

**Keywords:** stereotactic radiation therapy, stereotactic ablative radiation therapy, checkpoint inhibitor, immunotherapy, hepatocellular carcinoma, HCC

## Abstract

**Background:** Current treatments of unresectable hepatocellular carcinoma (HCC) are trans-arterial chemo-embolization (TACE), stereotactic body radiotherapy (SBRT), and targeted therapy. However, these treatments are limited in efficacy and safety for patients with large tumor sizes. Here, we report a case series of combined SBRT and anti-PD-1 therapy in patients with unresectable HCC of large tumors.

**Methods:** This is a retrospective case series of five patients with unresectable hepatocellular carcinoma who were treated with SBRT followed by anti-PD1 antibodies. Four patients (80%) received a single dose of TACE prior to SBRT. All patients had advanced HCC ineligible of curative intervention. In this study, we report their treatment responses according to modified RECIST (response evaluation criteria in solid tumor) criteria, 1-year local control (LC), progression-free survival (PFS), 1-year overall survival (OS) rate, and toxicities.

**Results:** Among the five evaluated patients, three patients had underlying diseases of hepatitis B and four patients had Barcelona clinic liver cancer stage C. The median size of their tumors was 9.8 cm (range: 9–16.1 cm). In addition, two patients had tumor vascular thrombosis and one had extra-hepatic disease. Five out of five patients (100%) responded to treatment, with two complete responses (CR) and three partial responses (PR). Among the partial responders, one had a down-staged tumor that became amenable for radiofrequency ablation for tumor clearance. No patient developed tumor progression at the time of analysis during the median follow-up of 14.9 months (range 8.6–19 months). The median PFS was 14.9 months (range: 8.6–19 months); 1-year LC and OS rate were both 100%. One patient had grade ≥ 3 toxicities (pneumonitis and skin reaction). There was no classical radiation-induced liver disease.

**Conclusions:** The results obtained from these 5 cases demonstrate impressive tumor control from the combination of SBRT and checkpoint inhibitors in patients with large tumors of advanced HCC. Further prospective trials are warranted.

## Introduction

Hepatocellular carcinoma (HCC) is one of the leading causes of cancer-related death globally (1). Liver resection, transplantation, or radiofrequency ablation (RFA) are the potentially curative therapies. However, over 70% of HCC are diagnosed as unresectable or advanced stage disease with limited effective therapies (2).

There are several loco-regional and systemic treatment options as recommended by the Barcelona Clinic Liver Cancer (BCLC) staging systems. Trans-arterial chemoembolization (TACE) is the most widely used loco-regional therapy (3, 4), however, its treatment efficacy is limited in sizable tumor or multi-focal disease with response rates of only 30% (5). For patients with more advanced diseases including either vascular invasion or distant metastasis, the recommended treatment is the small molecule multi-kinase inhibitor, Sorafenib or Lenvatinib. Unfortunately, its survival benefit is only modest and often associated with prohibitive side effects (6, 7). Therefore, novel therapeutic strategies for unresectable HCC patients are desperately needed.

HCC has been shown to be associated with inflammation and the immunosuppressive microenvironment (8). High expression of programmed death-ligand 1 (PD-L1) in tumors correlate with a poorer prognosis in patients with resected HCC (9). Up-regulation of programmed cell death protein-1 (PD-1) and PD-L1 on T cells is often associated with more advanced disease stages and higher risks of recurrence (10). These findings suggest that immunotherapy approaches may benefit HCC patients.

This premise was supported by two recently published phase II studies. In the CheckMate 040 study, the anti-PD-1 inhibitor Nivolumab has shown substantial tumor response (15–20%) with promising duration of response (median: 16 months), favorable survival, and manageable toxicity profile in advanced HCC patients who have previously received or were intolerant to Sorafenib (11). In the KEYNOTE-224 study, another anti-PD-1 inhibitor Pembrolizumab has reported similar findings (12). As such, both drugs have received accelerated approval in the USA for the treatment of HCC who were previously treated with Sorafenib.

Stereotactic body radiotherapy (SBRT) has emerged as a promising local treatment option for inoperable HCC with local control rates of 60–100% at 2 years (13–15). Recent data has shown that radiation could induce immunogenic cell death and convert the irradiated tumor into *in-situ* vaccines to prime the immune system (16). In addition, radiation could re-program the tumor stromal microenvironment against the immune evasion mechanisms of cancer (17). As a result, combined radiation and immunotherapy offers better local tumor regression and systemic (abscopal) control when compared to single modality treatments (18, 19). These findings have also been clinically reported at multiple disease sites, including case reports of lung cancer and melanoma (20, 21).

Herein, we report a clinical case series of the combined checkpoint inhibitor and stereotactic body radiotherapy for the treatment of unresectable, large HCC.

## Materials and Methods

### Patients

This is a retrospective study that was conducted at Queen Mary Hospital, the University of Hong Kong. Five patients who received combined SBRT and anti-PD-1 therapy for unresectable HCC from January 2017 to December 2018 were included. Patients had radiological diagnosis of HCC based on the typical pattern of enhancement and washout in multi-phasic computed tomography (CT) according to dynamic imaging criteria.

Patients who deemed unsuitable for curative surgical interventions were discussed in the multi-disciplinary tumor (MDT) board among hepato-biliary surgeons, radiation oncologists, medical oncologists, and interventional radiologists. Locally advanced tumors were defined as follows: tumor diameter >5 cm, number of lesions ≤3, or presence of intra-hepatic vascular invasion. Patients were offered the combined SBRT and anti-PD1 therapy as an experimental therapy or alternatively TACE, the standard of care. The recommendation was based on the poor historical outcomes achieved by TACE in this population (median OS of 6–11.8 months) (22), and driven by the promising anti-tumor activity of the checkpoint inhibitor as well as the potential synergistic effect between SBRT and immunotherapy. A total of 40 patients received radiation therapy during the study period, with 25 patients who had tumors >5 cm. Five of these patients agreed to the combined treatment, which was limited by the cost of the immunotherapy since the treatment was not covered by government insurance.

### Treatment

Patients with Child-Pugh (CP) A liver function (patients #2–5) received single doses of TACE followed by 5-fraction SBRT at 4 weeks. This was then followed by Anti-PD-1 inhibitor Nivolumab starting at 2 weeks upon completion of SBRT. Patient #1 presented with CP-B liver function and received single-fraction SBRT (8 Gy) followed by Nivolumab starting immediately at 2 weeks after SBRT. He later received another course of 5-fraction SBRT after improved hepatic function to CP-A. Patients with hepatitis B viral infection were covered with anti-viral therapy before study treatment.

#### TACE

TACE in our center was performed by supra-selective cannulation of the supplying tumor artery. The emulsion was prepared by mixing lipiodol with cisplatin in a 1:1 ratio using the pumping method, which was then slowly injected under fluoroscopic monitoring according to the size of the tumor and the arterial blood flow.

#### Radiotherapy

For SBRT planning, patients were immobilized via a vacuum foam bag (Vac-LokTM; MEDTEC, Iowa, USA) and active breathing control to reduce the amplitude of liver motion caused by breathing. Imaging was performed on the inhale breath-hold contrast computed tomography (CT). GTV was defined as tumor focus that was visualized on contrast imaging together with expansion to include the lipiodol stained area. The clinical target volume (CTV) was defined as GTV plus a margin of 0–5 mm. The individualized PTV margins were formulated to compensate the respiratory motion and set-up errors. Cone beam CT was acquired on board before each treatment. The tumor localization was then based on lipiodol retention when visible or as seen on the diaphragm as tumor surrogate. The dose was prescribed according to the Radiation Therapy Oncology Group (RTOG) 1112 protocol (23). The final dose was determined such that a maximum tumoricidal dose could be delivered to tumors while respecting the tolerance dose of organs-at-risk to the limits of RTOG1112. A total dose ranging from 27.5 to 35 Gy in 5 fractions (CP-A liver function) or 8 Gy in 1 fraction (CP-B liver function) followed by 30 Gy in 5 fractions were prescribed.

#### Anti-PD1 Therapy

Intravenous Nivolumab at a dose of 3 mg per kg was started at 2 weeks upon completion of SBRT and subsequently thereafter for every 2 weeks until disease progression, unacceptable toxicity, patient's refusal, or clinicians' decision.

### Data Collection and Outcomes

Patients were followed regularly according to our routine practice by the oncologists and surgeons in order to monitor adverse events and treatment responses. Blood tests were performed for complete blood count, renal and liver function, coagulation studies, and alpha-fetoprotein (AFP) every 2 weeks while on active treatment and every 3 months thereafter. Surveillance imaging was carried out every 3 months with contrast enhanced CT.

We reported the objective response rate (ORR) according to the Modified Response Evaluation Criteria for Solid Tumors (mRECIST). Furthermore, we reported progression-free survival (PFS), local control (LC), overall survival (OS), toxicities, and alpha-feto protein (AFP) response. PFS was defined as the period from the date of beginning treatment to the time of disease progression, which was censored at the last follow-up if the patient was still alive. LC was defined as the absence of progressive disease within the planning target volume (PTV). OS was calculated from the start of study treatment until the date of final follow-up or death. An AFP response was defined as a drop of at least 20% from baseline. Toxicities were graded using the National Cancer Institute Common Terminology Criteria for Adverse Events (CTCAE) version 5.0.

Continuous variables were presented as medians and ranges. Survivals were studied with the Kaplan-Meier method. Data was analyzed using R version 3.25 (Vienna, Austria).

## Results

### Patient Characteristics

Five patients were included in this case series. The median follow-up time was 14.9 months (range: 8.6–19 months), and all patients were alive during the last follow-up. Baseline patient characteristics are presented in Table 1. Median age was 78 years (range: 54–86 years) and all patients were male. Three patients (60%) were hepatitis B carriers. One patient (20%) had CP class B. Two patients (40%) were ECOG performance status of 2, two (40%) were ECOG 1, and one (20%) was ECOG 0. Median size of tumor was 9.8 cm (range: 8.5–16.1 cm). Median GTV volume was 451.7 cc (range: 257.6–1588.9 cc). Four (80%) had BCLC stage C disease. Two patients had portal vein invasion (40%) and one had lung metastases (20%). All patients were treatment naïve.

**Table 1:** Baseline tumor and patient characteristics.

**Patient**	**Age**	**Sex**	**Hepatitis B carrier**	**Tumor size (the greatest dimension in cm)**	**GTV volume (cc)**	**No of tumor**	**ECOG PS**	**CP score**	**BCLC stage**	**Intra-hepatic vascular invasion**	**Extra-hepatic vascular invasion**	**Metastasis**	**AFP level (nmol/L)**
1	66	M	No	16.1	1288.9	1	1	B8	C	Yes (left PV)	No	Yes (lung)	6,553
2	78	M	No	12.8	1140.1	1	1	A6	C	Yes (right PV)	No	No	628
3	86	M	Yes	9.8	451.7	1	2	A5	C	No	No	No	197
4	78	M	Yes	8.5	318.7	1	2	A6	C	No	No	No	3
5	54	M	Yes	9.0	257.6	1	0	A5	A	No	No	No	30

*GTV, gross tumor volume; ECOG PS, Eastern Cooperative Group Performance Status, CP score, Child-Pugh score; AFP, alpha-feto protein, PV, portal vein; intrahepatic vascular invasion includes invasions to intra-hepatic portal vein branch, left or right portal vein, and main hepatic vein; extra-hepatic vascular invasion includes invasions to the main portal vein and inferior vena cava*.

### Treatment Characteristics

**Figure 1:**
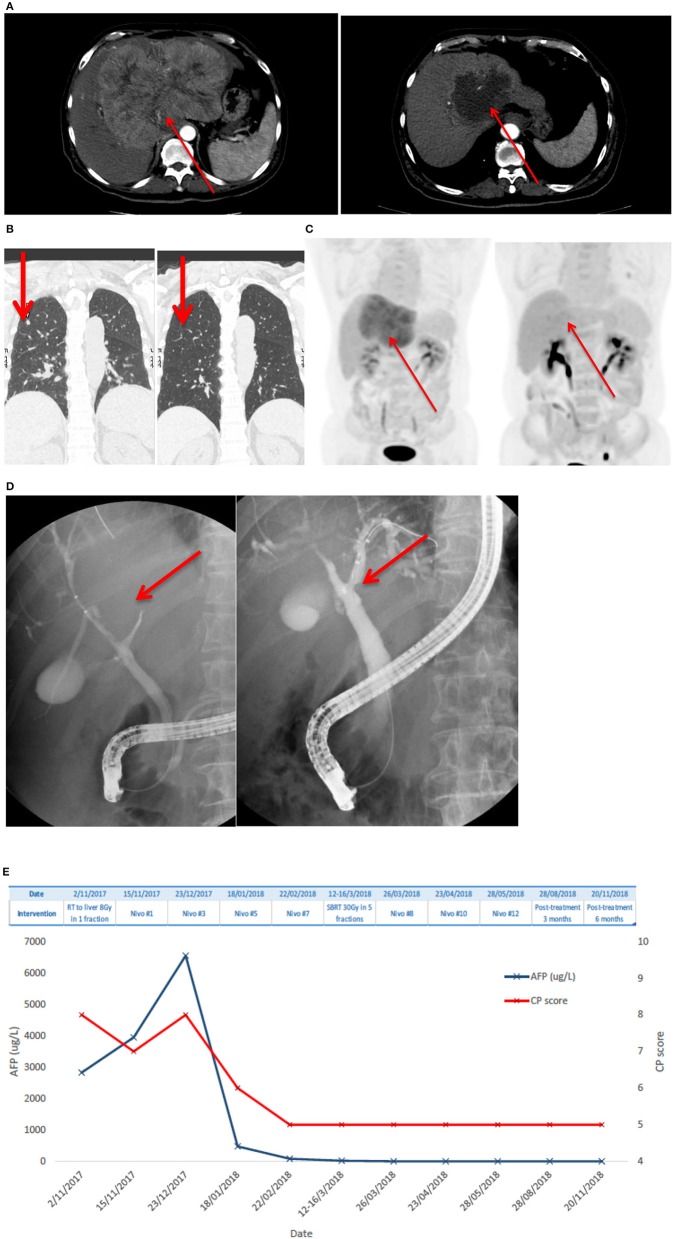
Patient #1 had advanced HCC and lung metastases who achieved complete remission after one single dose of 8 Gy SBRT and anti-PD1 antibody, followed by additional dose of 30 Gy in 5 fractions SBRT. **(A)** Shows a large arterial enhancing HCC over the left lobe of liver at diagnosis; there is marked reduction of tumor size and enhancing component of the lesion after radiotherapy and Nivolumab. **(B)** Demonstrates the complete resolution of lung metastases. **(C)** Shows that PET-CT revealed complete metabolic resolution of HCC. **(D)** Shows a compressed left main hepatic duct with minimally visible peripheral ducts on endoscopic retrograde cholangio-pancreatography (left) and resolved compression with fully visible ductal-branches after the treatment. **(E)** Shows AFP level and CP scores at different time points. AFP level improved and CP score improved over time.

Patient #1 received SBRT (8 Gy in 1 fraction) upfront in view of CP score 8 and uninvolved liver of <700 ml. After six cycles of Nivolumab with improvement of liver function to CP score A5 and a reduction in tumor size, another course of SBRT (30 Gy in 5 fractions) was administered. Nivolumab was resumed 2 weeks afterwards and stopped after a total of 12 cycles based on the clinicians' decision on complete tumor eradication. Details of treatment outcome of patient #1 are shown in Figure 1.

Patient #2–4 were treated with TACE and SBRT followed by Nivolumab with a median of 7 cycles (range: 2–19 cycles). All three of these patients stopped Nivolumab due to cost issues. Similarly, patient #5 received TACE and SBRT followed by Nivolumab. This patient developed a grade 3 skin reaction during the first cycle and was subsequently given Pembrolizumab for three more cycles. Treatment stopped after he developed grade 3 pneumonitis.

Patients #1 and #3 received no subsequent treatment, Patients #2 and #4 were given Lenvatinib, and Patient #5 had radio-frequency ablation (RFA) to residual lesion (1.4 cm) after initially excellent treatment response to limited cycles of immunotherapy. Treatment details of each patient are also summarized in Table 2.

**Table 2:** Treatment details, outcome, and toxicities of patients.

**Patient**	**Tumor size at the last follow-up (cm)**	**Degree of Lipiodol uptake^#^**	**Reduction in tumor size (RECIST response)**	**Imaging response assessment according to mRECIST (24)**	**AFP response**	**Treatment regimen**	**Worst toxicity**	**PFS (months)**	**Follow-up (months)**	**Status**
1	4.8	NA	70.7% (PR)	CR	Yes	Two courses of **SBRT** 8 Gy in 1 fraction, 30 Gy in 5 fractions, and total **12 cycles of Nivo**	Grade 2 raised ALT/AST (Nivo)	19.0	19.0	Alive
2	8.9	Moderate	30.5% (PR)	PR	Yes	TACE followed by **SBRT** 27.5 Gy in 5 fractions, and total **10 cycles of Nivo**	Grade 1 raised ALT/AST and grade 1 fatigue (Nivo)	17.2	17.2	Death (aspiration pneumonia)
3	5.9	Moderate	38.7% (PR)	PR	Yes	TACE followed by **SBRT** 32.5 Gy in 5 fractions, and total **19 cycles of Nivo**	Grade 1 raised ALT (Nivo)	14.9	14.9	Alive
4	5.6	Moderate	36.4% (PR)	CR	NE^*^	TACE followed by **SBRT** 35 Gy in 5 fractions, and total **2 cycles of Nivo**	Grade 2 raised ALT/AST and grade 2 fatigue (Nivo)	11.0	11.0	Alive
5	1.4	Intense	84.4% (PR)	PR	Yes	TACE followed by **SBRT** 35 Gy in 5 fractions, then **Nivo** **×** **1 cycle and Pembro** **×** **3 cycles**	Grade 3 skin reaction (Nivo); Grade 3 pneumonitis (Pembro)	8.6	8.6	Alive

*RECIST, response evaluation criteria in solid tumor, mRECIST, modified response evaluation criteria in solid tumor; AFP, alpha-feto protein; PFS, progression-free Survival; CR, complete response, PR, partial response, SD, static disease; NE, not evaluable; TACE, trans-arterial chemo-embolization; SBRT, stereotactic body radiotherapy; Nivo, Nivolumab; Pembro, Pembrolizumab; ALT, alanine transaminase; AST, aspartate transaminase*.

# *Degree of lipiodol uptake was assessed in venous phase CT scan in 3 weeks after TACE and categorized as (1) Complete (100% of tumor volume), (2) intense (>75% of tumor volume), (3) moderate (≤75% of umor volume), and (4) low (patchy deposit only)*.

* *Not evaluable as the baseline AFP level was normal*.

### Outcome

**Figure 2:**
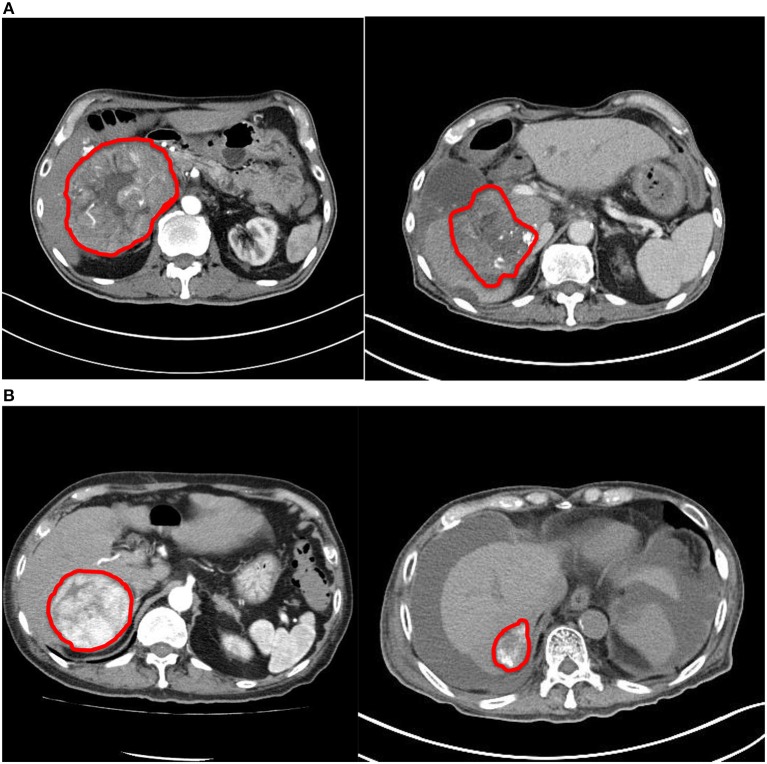
Comparison of computed tomography images before and after treatment. **(A)** Patient #2. **(B)** Patient #4. Both tumors demonstrated significant reduction in size and enhancing component after treatment.

The ORR was 100%: of them, two (40%) had complete response (CR) and three (60%) had partial response (PR) (Figures 1, 2). The median reduction of tumor diameter was 38.7% (range: 30.5–84.4%).

At the time of analysis, four patients were still alive, although one died of aspiration pneumonia at 17 months from the date of starting treatment; No disease progression was noted. The median PFS was 14.9 months (range: 8.6–19 months). The 1-year local rate and overall survival were both 100%. AFP responses were observed in all four patients (100%) with baseline marker >20 nmol/L.

One patient (patient #5) had Grade ≥ 3 toxicities with the development of pneumonitis and skin reaction. No patient developed classic radiation-induced liver disease (RILD) or CP score progression ≥2 at 3 and 6 months. Yet, patient #4 initially presented with CP-A6 and noted deterioration of CP score to B7 with development of ascites, which was controlled with medical treatment at 6–9 months after SBRT. The liver function gradually improved afterwards. No reactivation of hepatitis B viral infection occurred.

## Discussion

To the best of our knowledge, this is the first reported study that has combined TACE, SBRT, and checkpoint inhibitor in unresectable locally advanced HCC. The 1-year local control rate of 100% is promising; the survival outcome is remarkable for this patient population, which is otherwise characterized by almost no effective options for treatment. Our preliminary findings have shown that SBRT combined with anti-PD-1 therapy is effective in treatment of this naïve patient population. SBRT may augment the anti-tumor activity of anti-PD1 therapy without unexpected toxicity.

Anti-PD-1 therapy is a clear breakthrough in advanced HCC treatment, however, only a minority of patients experience durable response (11, 12). In CheckMate-040 and Keynote-224 studies, the anti-PD-1 therapy resulted in an ORR of 14–17% among advanced HCC patients who had previously received Sorafenib (11, 12). The anti-tumor activity was durable among the responders, which led to favorable survival outcomes compared to historical data with median OS ranging from 12.9 to 15.6 months. Among 80 patients who were Sorafenib-naïve in the Checkmate study, Nivolumab was associated with an ORR of 20% and 12-month OS rate of 75%. Current efforts are now making progress in focusing on various combined approaches to increase the effectiveness of treatment.

The 1-year local control rate of 100% in our series was compared favorably to that of previous SBRT series in similar populations (15, 25). Bujold et al. showed a 1-year local control rate of 87% in 102 patients with tumor median size of 7.8 cm (range: 1.4–23.1 cm) (15). Gkika et al. reported a 1-year local control rate of 77% in 47 patients with median size of 7 cm (range: 5–10 cm) (25). The larger median tumor size of 9.8 cm (range 8.5–16.1 cm) and lower radiation dose used in our study (though with a limited number of patients) implied that an even more impressive local control could be achieved by the combined strategy. Prospective studies with larger samples of patients are warranted.

The 1-year OS rate of 100% was promising against our institution's historical data of 36.5% in similar populations (26). Our patient cohort of locally advanced HCC treated with TACE as the traditional standard of care reported a median OS of only 6–11.8 months (22). In the current report, there were two complete radiological responders (patient #1 and #4); the complete clearance of the tumor was further confirmed by liver biopsy in patient #1. Patients remained disease-free without treatment at 8 and 12 months, and survived 11 and 19 months, respectively.

The combined strategy seemed to be highly effective in tumor shrinkage. Achieving tumor down staging may facilitate the subsequent use of curative therapy, which in turn further improves survival. Notably, patient (#5) had good shrinkage of tumor (PR) after 4 months of treatment; RFA to the residual tumor was done as a curative procedure with interval CT suggesting complete ablation of the tumor. The promising potential of this strategy as a down-staging therapy remains to be developed.

Despite findings in previous studies that have shown that SBRT is an effective local treatment in high-risk HCC, failure outside the irradiated area is concerningly common. Therefore, the survival benefit of radiation remains controversial (15, 25). Notably in the present series beyond the 1-year LC rate of 100%, no patient developed out-of-field progression. Only one patient had lung metastases, which was completely eradicated. We postulated that the checkpoint inhibitor works synergistically with SBRT to enhance the systemic clearance of occult or overt metastases. Theoretically, if out-of-field areas are better controlled with improved systemic therapy, then the local control with SBRT would become more important.

It should also be noted that the safety profile of anti-PD-1 therapy in combination with SBRT in this population was consistent with that of mono-therapy in previously reported studies. One patient developed grade 3 pneumonitis and skin reaction after the checkpoint inhibitor, which are known adverse effects. The most common hepatic adverse event was low-grade elevation of enzymes. Overall, there were no unexpected safety signals of anti-PD-1 therapy after SBRT. Yet, analysis of the incidence rate of adverse events was limited by the small sample size.

This study is limited by case reports of small sample size, short follow-up times, and a single institutional experience. Also, the potential selection bias in this retrospective series should not be overlooked, as immunotherapy was expensive and only those with good financial status were able to afford it. Furthermore, the substantial heterogeneity of patient population, treatment regimen, and radiation dose prescription in the current series may affect the interpretation of our findings. For example, the role of second course SBRT after initial response of immunotherapy and 8 Gy single fraction radiotherapy was unclear in patient #1. The limited, promising findings for this population of patients with expected dismal outcomes mobilizes future clinical trials and research on this topic.

In summary, the present study provides a rationale to conduct a prospective trial with an adequately powered sample size to test the efficacy of combined SBRT and checkpoint inhibitor for the unresectable locally advanced HCC population. Such a study was recently developed in our center (NCT03817736). Future studies may focus on more effective plans of each modality and an optimal “concerted” combination. Furthermore, translational work is also needed to better characterize the potential immune activation properties of SBRT in order to optimize the schedule and radiation dose fractionation of this combination therapy.

## Data Availability Statement

All datasets generated for this study are included in the article/supplementary material.

## Ethics Statement

The Institutional Review Board of The University of Hong Kong/Hospital Authority Hong Kong West Cluster (HKU/HA HKW IRB) has performed ethics approval to this study, with the IRB Reference Number: UW 19-286. Written informed consent to participate for all patients are obtained.

## Author Contributions

C-LC and AC: conception and design. C-LC and KC: collection and assembly of data. C-LC, AC, KC, and F-MK: data analysis and interpretation, manuscript writing, and final approval of manuscript.

### Conflict of Interest

The authors declare that the research was conducted in the absence of any commercial or financial relationships that could be construed as a potential conflict of interest.
